# Border-associated macrophages in the central nervous system

**DOI:** 10.1186/s12974-024-03059-x

**Published:** 2024-03-13

**Authors:** Rui Sun, Haowu Jiang

**Affiliations:** 1 Department of Neurological Surgery, Washington University School of Medicine in St. Louis, 660 S. Euclid Ave., Box 8057, St. Louis, MO 63110 USA; 2 Department of Anesthesiology, Washington University Pain Center, Washington University School of Medicine in St. Louis, 660 S. Euclid Ave., CB 8054, St. Louis, MO 63110 USA

**Keywords:** Brain, Central nervous system, Border-associated macrophages, Microglia, Neurodegeneration, Cancer

## Abstract

Tissue-resident macrophages play an important role in the local maintenance of homeostasis and immune surveillance. In the central nervous system (CNS), brain macrophages are anatomically divided into parenchymal microglia and non-parenchymal border-associated macrophages (BAMs). Among these immune cell populations, microglia have been well-studied for their roles during development as well as in health and disease. BAMs, mostly located in the choroid plexus, meningeal and perivascular spaces, are now gaining increased attention due to advancements in multi-omics technologies and genetic methodologies. Research on BAMs over the past decade has focused on their ontogeny, immunophenotypes, involvement in various CNS diseases, and potential as therapeutic targets. Unlike microglia, BAMs display mixed origins and distinct self-renewal capacity. BAMs are believed to regulate neuroimmune responses associated with brain barriers and contribute to immune-mediated neuropathology. Notably, BAMs have been observed to function in diverse cerebral pathologies, including Alzheimer’s disease, Parkinson’s disease, multiple sclerosis, ischemic stroke, and gliomas. The elucidation of the heterogeneity and diverse functions of BAMs during homeostasis and neuroinflammation is mesmerizing, since it may shed light on the precision medicine that emphasizes deep insights into programming cues in the unique brain immune microenvironment. In this review, we delve into the latest findings on BAMs, covering aspects like their origins, self-renewal capacity, adaptability, and implications in different brain disorders.

## Introduction

Macrophages are known to be phagocytes of the innate immune system. They not only perform a defensive function against invading pathogens such as bacteria and viruses, but also help maintain immune homeostasis through the removal of apoptotic or necrotic cells in the body [1]. Macrophages take part in both innate and adaptive immune responses involved in development, inflammation, tissue repair, and immunological memory [2, 3]. Macrophages express a variety of sensors such as scavenger receptors, integrins, and Toll-like receptors, enabling them to detect and respond to a wide range of environmental stimuli [1, 2]. Macrophages utilize multifaceted mechanisms to fulfill their immunomodulatory functions. These include phagocytosis; the release of diverse inflammatory mediators such as cytokines, chemokines, bioactive lipids, enzymes, reactive oxygen and nitrogen species, membrane-enclosed vesicles, and certain metabolites; the expression of co-stimulatory molecules such as CD40 and PD-L1; and triggering a T cell response through antigen processing and presentation [4, 5].

Macrophages are found in every tissue and exhibit tissue-specific functions. Therefore, tissue-resident macrophages are extremely heterogeneous and phenotypically distinct. They are also dynamic and adaptive. Work from research over the last decade has revealed that macrophages can be epigenetically reprogrammed to react to both physiological and danger signals from the tissue microenvironment [3, 4, 6]. As a result, macrophages demonstrate both tissue and disease state-associated characteristics, contributing to tissue remodeling, host defense, wound healing, and immune modulation. As the developmental and disease-relevant heterogeneity of tissue macrophages unravels, macrophages residing in the central nervous system (CNS) have become a focus of attention. The CNS has been considered unique for being immune-privileged, as the blood–brain barrier (BBB) acts as a roadblock, preventing microorganism entry and the influx of circulating immune cells [7]. CNS homeostasis is principally maintained by brain-resident macrophages. Notably, in physiological conditions, brain macrophages are anatomically classified as microglia in the brain parenchyma, and non-parenchymal border-associated macrophages (BAMs) which are located at the blood–brain and blood-CSF (cerebrospinal fluid) barriers as well as in the meninges [8, 9]. Microglia serve as the most abundant phagocytes in the adult brain, accounting for approximately 10% of total cells [10, 11]. Furthermore, microglia have been the subject of study since their discovery in 1919 [12]. Until now, historic breakthroughs in investigations of microglia have included their ontogeny and self-maintenance during homeostasis [8, 13], physiological regulations such as synapse pruning and myelin turnover [14, 15], and functions in the context of neurodegenerative and neuropsychiatric diseases, as well as traumatic brain injury [8, 13, 16–18]. Research on BAMs began much later than on microglia but has progressed rapidly owing to new technologies, such as mass cytometry, fate-mapping, single-cell RNA sequencing (scRNA-seq), in vivo imaging, and Cre recombinase-mediated mutagenesis [19]. Emerging evidence particularly indicates that BAMs, compared to microglia, function differently in neuropathology [20]. In this review, we aim to summarize recent advances in BAMs, focusing on their ontogeny, self-maintenance, and roles in normal neurodevelopment and different CNS disorders.

## The macrophage system

Macrophages were first identified by Metchnikoff as a type of responding cells with phagocytic activity [21]. Later, it was presumed that they originated from the reticuloendothelial system, which supports the generation and differentiation of vascular endothelial cells [22]. This early concept, which posited that macrophages originated from tissue, was then challenged, as advancing experimental methods provided evidence that a large group of macrophages were derived from circulating monocytes in the blood [23, 24]. Subsequently, this led to the establishment of a broader definition describing distinct macrophage subsets in all tissues: the mononuclear phagocytic system (MPS) [24]. MPS encompasses all terminal-differentiated and intermediate phagocytic cells, along with their progenitors. It proposes a linear pattern where bone marrow-resident precursors evolve into blood monocytes as an intermediate state, which then migrate and differentiate into specialized macrophages in various organs [24].

In adult mammals, macrophages display remarkable morphological and functional diversity in multiple organs, such as the brain, liver, lung, spleen, kidney, skin, and adipose tissues. It was believed that in these organs, definitive organizational structures dictated the differentiation process of specialized macrophages, and that hematopoiesis in bone marrow supported the generation of their common progenitors. However, this evolutionary trajectory of tissue-resident macrophages came into question when several studies discovered that some tissue-resident macrophages had the capacity of self-renewal and exhibited local proliferation under certain circumstances [25–27]. In addition, it was observed that the resident peritoneal macrophages in mice could survive for a long period in a steady state without replacement by recruited blood monocytes, which raised the possibility of a dual origin for some tissue-resident macrophages [28]. Subsequently, developed techniques, including immunohistochemistry, flow cytometry, DNA microarray, and fate-mapping/lineage tracing with genetically modified mice, gradually revealed that during ontogeny, primitive macrophage populations derived from the embryonic yolk sac or fetal liver spread into entire peripheral tissues, colonize, and maintain themselves in tissues by self-renewal. These populations are the sources of some tissue-resident macrophages in adulthood, such as microglia and Langerhans cells [29–36]. Finally, all these discoveries led to an improved notion of the in vivo macrophage system. In fetal development, macrophage precursors from the yolk sac and fetal liver migrate and settle down in all tissue rudiments, constituting the original tissue macrophages with self-proliferative capacities. Into adulthood, macrophages derived from hematopoietic stem cells in bone marrow, with blood monocytes as the intermediate cell-type, replenish most of the tissue-resident macrophage pools within the body [37]. However, there are some exceptions. For instance, brain-resident microglia are derived solely from macrophage precursors in the yolk sac and repopulate in the CNS throughout life [33, 34]; Langerhans cells in the epidermal layer of the skin, originating embryonically, remain independent of bone marrow-derived precursors in the steady state [38–42].

## Brain macrophages

**Fig. 1:**
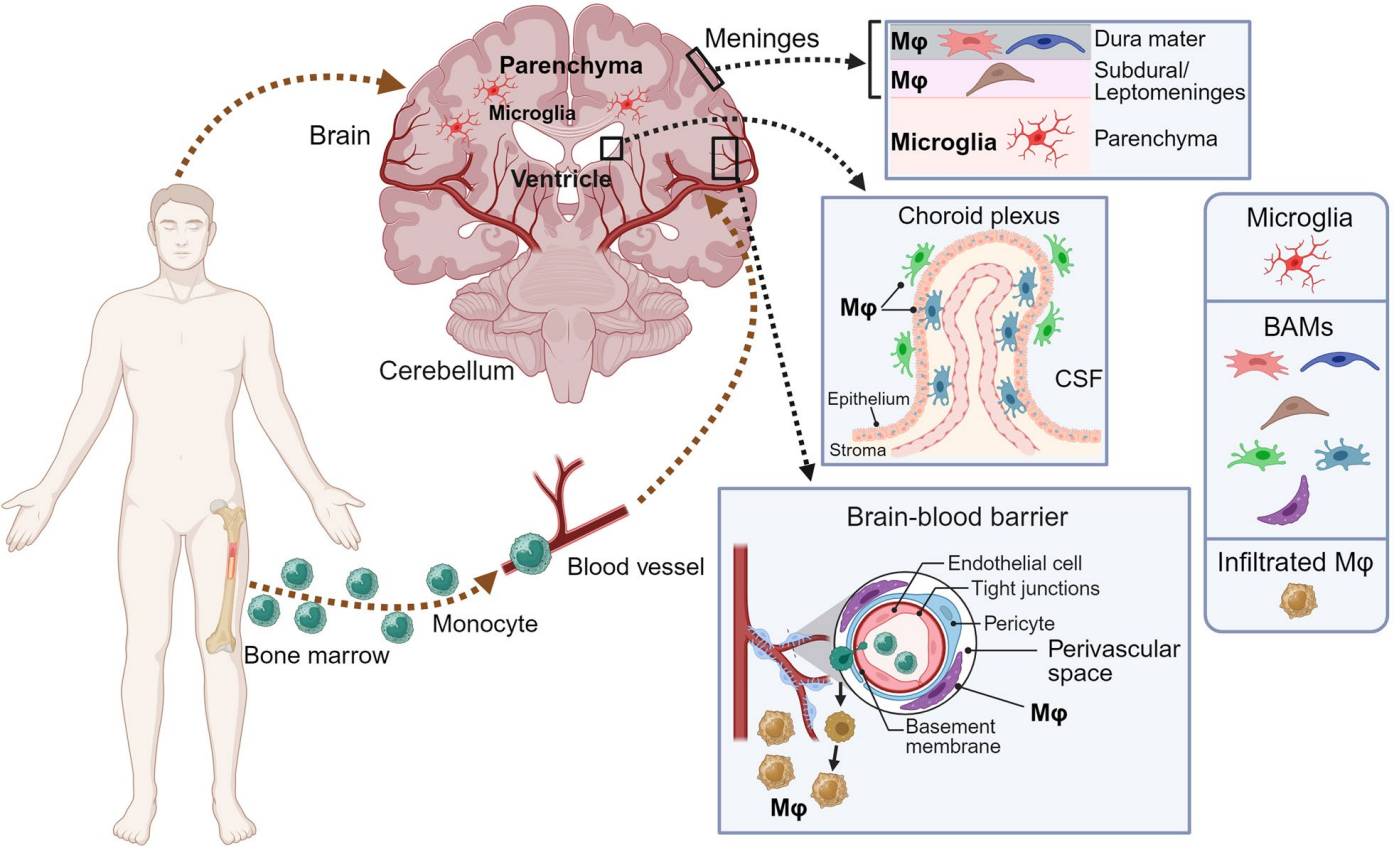
Distinct macrophage subsets in the central nervous system. Parenchyma: microglia; Brain-circulation interface: macrophages located in the meninges, choroid plexus, and perivascular spaces; Migrated from blood: monocytes that migrate into the brain from a dysregulated brain–blood barrier and differentiate into macrophages. *BAMs* border-associated macrophages, *CSF* cerebrospinal fluid, *MФ* macrophages

To solve the mystery of the CNS immune system, extensive studies on brain macrophages have been undertaken for many years. Brain macrophages are highly heterogeneous, comprising resident populations including microglia and BAMs, and infiltrating monocyte-derived macrophages (MDMs) under physiological and disease conditions. Until recently, research utilizing transgenic mouse models and high-throughput sequencing technologies has revealed that these distinct macrophage subsets (Fig. 1), corresponding to their origins, exert differential functions in both brain homeostasis and pathogenesis [43–48].

Ontogenetically, microglia exclusively arise from early myeloid progenitors in the embryonic yolk sac, which move to the CNS and differentiate into microglia [33, 34]. Microglia maintain their population in the brain by self-renewal and show different functional states through regulating their proliferation and phenotypes [8, 10, 11, 13–18]. Compared to microglia, BAMs exhibit heterogeneous ontogenies and are essentially comprised of different macrophage subsets, including meningeal macrophages, perivascular macrophages (PVMs), and choroid plexus macrophages [9, 48–50]. During embryonic development, both yolk sac- and fetal liver-derived progenitors contribute to the BAM pool, and they sustain via the clonal expansion at brain border structures throughout life [37, 48–50]. BAMs can be divided into more detailed subpopulations based on their anatomical sites: subdural/leptomeningeal macrophages (sdΜΦ), dural macrophages (dmΜΦ), stromal choroid plexus macrophages (cpΜΦ), choroid epiplexus macrophages (cp^epi^ΜΦ), and PVMs [49, 50]. The sdΜΦ populate the pia mater and the dmΜΦ are located at the dura mater of the meninges. Choroid plexus macrophages reside in the stromal space between the epithelial and endothelial layers (cpΜΦ) and along the apical epithelial surface (cp^epi^ΜΦ, “Kolmer cells”) [9, 49–51]. PVMs are primarily found surrounding cerebral vessels. Of note, the maintenance of PVMs around the BBB and sdΜΦ in the leptomeninges does not depend on circulating monocytes in the blood. They can subsist over a long period and are thereby known as long-lived macrophages [48, 50]. Intriguingly, the dmΜΦ have shown a dual origin: one subpopulation expressing major histocompatibility complex class II (MHCII) emerges from childhood, revealing its recruitment from bone marrow; the other subset which lacks MHCII expression presents the long-lived characteristic, originating from embryonic progenitors [48, 49]. A recent investigation revealed that, under homeostasis, a group of monocytes in the brain and spinal dural meninges originates not from the blood, but directly from the adjacent skull and vertebral bone marrow [52]. In the choroid plexus, both cpΜΦ and cp^epi^ΜΦ, which are located around the blood-CSF barrier (BCB),derive from primitive macrophages during embryogenesis. However, the cpΜΦ are constantly replenished by CCR2^+^Ly6C^high^ monocytes from the bloodstream throughout adult life, rather than through self-renewal [48–51]. Thus, in comparison to PVMs and sdΜΦ, which have minimal turnover, the cpΜΦ in the choroid plexus demonstrate a relatively short lifespan due to their steady turnover by blood monocytes [49–51]. Similar to microglia, these subsets of BAMs display distinct transcriptional profiles in both healthy and diseased conditions [53–55]. Their tissue-specific functions warrant thorough exploration in future research.

In the CNS, recruited monocytes and MDMs stem from the hematopoietic system in the bone marrow. After birth, in a steady state, hematopoietic stem cells (HSCs) continue to differentiate into Ly6C^+^ monocytes and exit the bone marrow niche [56, 57]. Ly6C^+^ monocytes, expressing CCR2, can rapidly traffic into tissues and lymph nodes or transform into blood-resident Ly6C^−^ monocytes [36, 58]. Postnatal Ly6C^+^ monocytes can mature into tissue-resident macrophages in certain organs, such as brain (choroid plexus), skin, lung, intestine, and heart, where they are capable of self-proliferation [41, 42, 48, 50, 51, 59–61]. During inflammation or injury, local tissue debris or pro-inflammatory mediators can continuously attract circulating monocytes to migrate into the tissue. Once in the inflamed tissue, monocytes are cued to secrete more inflammatory mediators, accelerating their accumulation and maturation into macrophages [57]. Apparently, the phenotypes of MDMs in different body positions are largely determined by the local environment and the stage in the inflammatory process. During aging, there is an increase in the proportion of MDMs in the CNS, a trend that is more evident in animals with neurodegenerative diseases [45]. Yet, macrophages in aged organisms exhibit more pro-inflammatory signatures compared to those in healthy, young adults. This includes reduced autophagy and phagocytosis, and increased secretion of IL-6 and TNFα [62, 63]. Furthermore, studies have shown evidence that aging impacts the BBB integrity, characterized by increased leakage and susceptibility to breakdown [64–67]. The damaged BBB offers opportunities for patrolling monocytes in the cerebrovascular system to infiltrate into the brain parenchyma. Accordingly, among brain-resident macrophage populations, the density of MDMs is at least partially regulated by the organism’s age. In addition, during aging, blood-borne macrophages tend to induce inflammation in the brain.

To date, microglia and BAMs are considered key players in controlling brain development, homeostasis, and neurodegenerative disorders such as Alzheimer's disease (AD) and Parkinson’s disease (PD) [19, 43–45]. However, blood-borne macrophages have been shown to migrate and vastly accumulate in the brain parenchyma in certain neuroinflammatory diseases. These include multiple sclerosis (MS) [68], ischemic stroke [69], traumatic brain injury [70], gliomas [71–73] and certain brain infections [74, 75]. Thus, it has been proposed that in CNS diseases, brain-resident macrophages contribute to the clearance of various debris and the resolution of inflammation, whereas blood-borne phagocytes play a pivotal role in driving cerebral immunopathology [20]. Although empirically determining which specialized macrophage population is driving or suppressing immune effector function in different disease contexts remains challenging, relevant studies are increasingly emerging. Notably, using a mouse model of heterotopic transplantation of bone marrow into brain parenchyma, a study has revealed that graft-derived macrophages in the brain display distinct responses to peripheral endotoxin challenge compared to microglia, although they have exhibited significant microglial characteristics, such as ramified morphology, longevity, clonal expansion, and radio-resistance [76]. Furthermore, over time in the CNS niche, the transcriptomes and chromatin accessibility landscapes of bone marrow-derived macrophages remain distinct from those of host microglia [76]. These results suggest that different brain macrophage subsets inherently impact disease progression in distinct ways. In a neuroinflammatory mouse model infected by *Trypanosoma brucei*, a species of parasites that invades the brain through its borders, researchers have revealed that brain-resident macrophages, including microglia and BAMs, initiate the initial immune defense and subsequent migration of blood monocytes across disrupted brain barriers. However, as the disease progresses, MDMs expand greatly and eventually outnumber the resident macrophages [20, 77]. These MDMs exhibit greater transcriptional plasticity and antimicrobial features, which lead to exacerbated inflammation as well as effective parasite killing [77]. Upon disease resolution, microglia progressively revert to a homeostatic state, while the recruited macrophages are rapidly cleared from the brain parenchyma [77]. Notably, BAMs, in contrast to disease-associated microglia (DAM), exhibit long-term transcriptional alterations [77], highlighting their unique functions in CNS disorders. Likewise, to systematically delineate brain macrophages, a study utilized scRNA-seq to interrogate the heterogeneity of myeloid cells in aging brains and murine models of AD [45]. Interestingly, the study has shown that the previously identified DAM in AD brains actually consist of two ontogenetically and functionally distinct cell populations. These include the triggering receptor expressed on myeloid cells 2 (TREM2)-dependent DAM, which exhibit a neuroprotective signature, and monocyte-derived disease inflammatory macrophages (DIMs) that typically accumulate in the brain during aging [45, 62]. Compared to healthy brains, the number of DIMs increases in AD brains and their function is independent of TREM2 [45]. In conclusion, a better understanding of the differential roles of brain macrophage subpopulations, such as BAMs, will aid in developing targeted therapeutic strategies and precision medicine for neurodegenerative and neuroinflammatory diseases.

## Border-associated macrophages

**Fig. 2:**
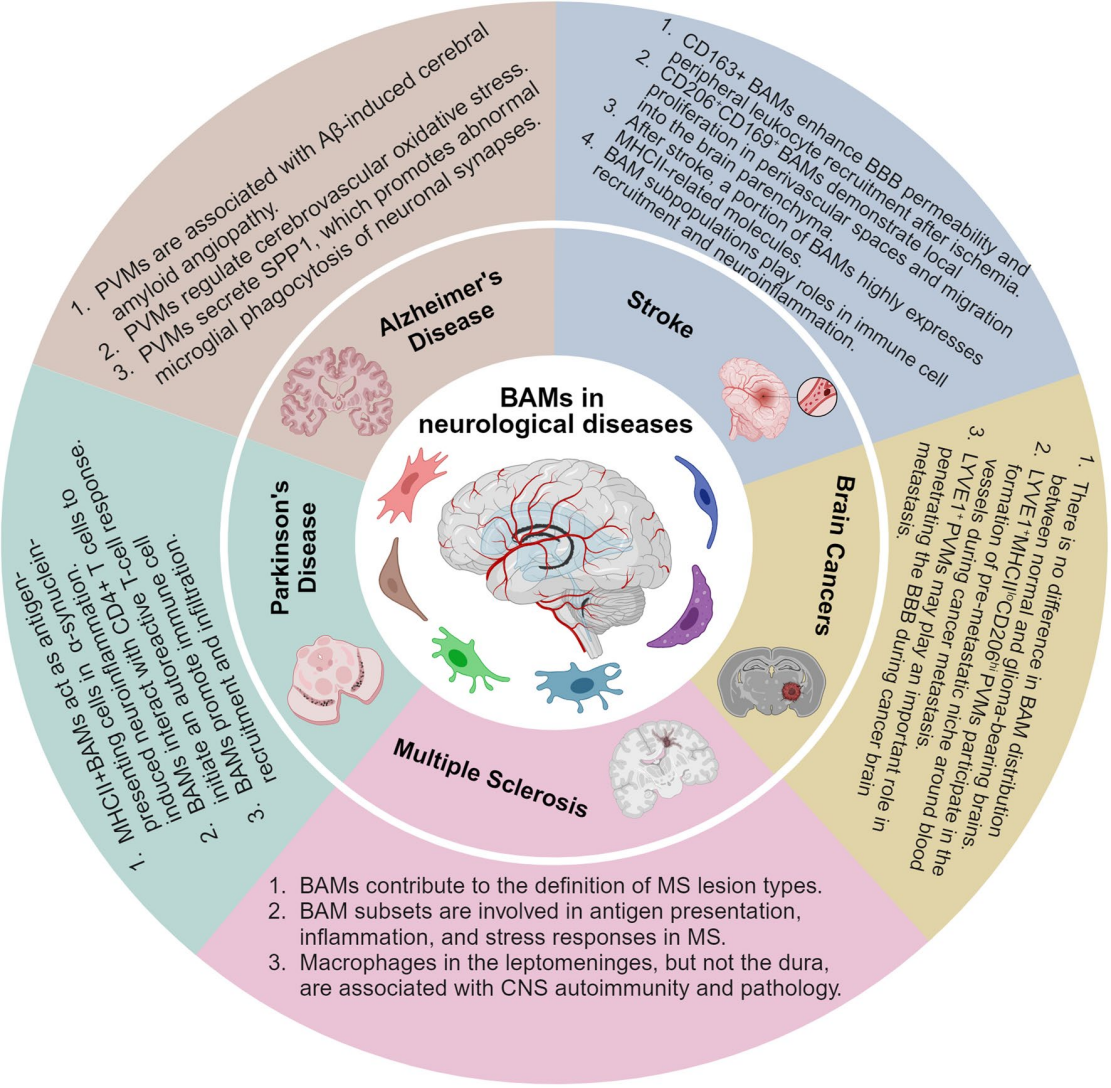
BAMs are involved in diverse cerebral pathologies. *BAMs* border-associated macrophages, *PVMs* perivascular macrophages, *MS* multiple sclerosis, *CNS *central nervous system

BAMs are receiving more attention since they have been observed to function in diverse cerebral pathologies [19], including AD [78], PD [79], MS [80], ischemic stroke [46], and gliomas [44]. Unlike microglia, BAMs show mixed ontogenies and distinct self-renewal capacity following experimental depletion and repopulation [49]. We now understand that BAMs comprise several anatomical subpopulations, including sdΜΦ, dmΜΦ, cpΜΦ, cp^epi^ΜΦ, and PVMs. Their phenotypes and potential involvement in CNS diseases are gradually being revealed through high-dimensional resolution techniques such as mass cytometry and scRNA-seq, bulk RNA-sequencing, fate-mapping, and microscopy [49]. Herein, we have summarized current data on the characteristics of BAMs (Table 1) and their roles in various neurological diseases (Fig. 2 and Table 2).

**Table 1:** Characteristics of brain-resident microglia and BAMs

Subset		Origin	Turnover	Morphology	Motility	Cell markers	Transcriptional profiles		References
Microglia		Embryonic yolk sac	Self-renewal	Homeostasis: ramified; Inflammation: amoeboid; Brain injury: bipolar rod	Cell bodies: stationary; Processes: actively mobile	SIGLEC-H, SALL1, TMEM119, P2RY12	*Tmem119*, *Siglech*, *Slc2a5*, *P2ry12*, *Sparc*, *Fcrls*, *Olfml3*, *Sall1*, *Hexb*, *Trem2*		[33, 34, 50, 53, 81–83, 91, 96–99, 106]
BAMs	sdΜΦ	Yolk sac; fetal liver;	Self-renewal	Homeostasis: elongated; Inflammation: extended protrusions	Cell bodies: partial motility; Processes: highly dynamic	CD206, CD38, CD169, CD163, CD36, LYVE1	*Lyve1*, *P2rx7*, *Ccr1*, *Egfl7*	*Mrc1*, *Cd163*, *Cd169*, *Cd36*, *Apoe*, *Ms4a7*, *Ms4a6c*, *Stab1*, *Lyz2*, *Pf4*, *Cbr2*, *Tgfbi*	[45, 48–50, 52, 84, 85, 91]
	dmΜΦ	Yolk sac; fetal liver	Self-renewal; skull and vertebral bone marrow	Homeostasis: pleomorphic, bipolar with many dendrites;					
	PVMs	Yolk sac; fetal liver	Self-renewal	Homeostasis: elongated; Inflammation: extended dendritic processes	Cell bodies: stationary; Processes: constantly extending and retracting their protrusions		*Mrc1*, *Cd163*, *Lyz2*, *Pf4*, *Lyve1*		[46–48, 50, 88, 89, 91, 104, 106, 110]
	cpΜΦ	Yolk sac; fetal liver	Self-renewal; blood monocyte	Embryonic: amoeboid with small processes; Adult: cpΜΦ, stellate with long, thin processes; cp^epi^ΜΦ, an amoeboid shape	Cell bodies: non-motile; Processes: highly dynamic		*Ccnd2*, *Ttr*, *Lilra5*		[48–51, 85–87, 90, 91, 105]
	cp^epi^ΜΦ	Yolk sac; fetal liver	Self-renewal		Cell bodies: high motility; Processes: highly dynamic		Significantly similar to microglia

**Table 2:** BAMs in distinct neurological diseases

Disease	Material	Potential roles of BAMs	References
Alzheimer’s Disease	1. TgCRND8 transgenic mice 2. Tg2576 transgenic mice	1. PVM turnover reduce cerebral amyloid angiopathy 2. PVMs express both NOX2 and CD36, which exacerbate the Aβ-induced oxidative stress 3. PVMs promote the expression of C1QA, GRN, and CTSB in microglia, leading to aberrant phagocytosis of neuronal synapses 4. PVMs may express APOE4, which is associated with neurovascular alterations and BBB breakdown	[78, 114–117, 121, 123–125]
Parkinson’s Disease	1. α-Synuclein overexpression in transgenic mice 2. Human postmortem brain tissues	1. MHCII^+^ BAMs present antigens to CD4^+^ T cells to initiate the anti-α-synuclein CD4^+^ T cell response 2. The depletion of BAMs reduces microglia activation and the recruitment of Ly6C^hi^ monocytes and CD4^+^ Th cells 3. BAMs in the meninges may contribute to the clearance of brain α-synuclein via the glymphatic system and meningeal lymphatic vessels 4. CD68^+^ BAMs have been observed to interact with CD3^+^ T cells in human brain tissues	[79, 132, 133]
Ischemic Stroke	1. A rat model of ischemia–reperfusion 2. MCAO mouse model 3. Post-mortem brain tissues of patients	1. CD163^+^ BAMs enhance leukocyte recruitment and BBB permeability via the production of VEGF after ischemia 2. Proliferated CD163^+^ BAMs can migrate to the brain parenchyma and induce inflammation 3. After ischemia, CD206^+^CD169^+^ BAMs show proliferation in perivascular spaces and subsequently accumulate in the brain parenchyma 4. MHCII^+^ BAM subsets may promote neuroinflammation by regulating adaptive immune responses, cellular oxidative phosphorylation, endocytosis, and immune cell recruitment	[104, 141–143]
Multiple Sclerosis	1. Human samples 2. EAE mouse model	1. BAMs are associated with MS lesion types and correlate with the pathology of MS 2. A subset of BAMs expressing *CD163*, *F13A1*, and *LYVE1* is involved in lesion inflammation and antigen presentation 3. Both sdΜΦ and PVMs in the leptomeninges participate in autoantigen presentation and the activation of autoreactive effector T cells 4. PVMs continue to proliferate during the chronic phase of MS	[80, 150, 151, 154, 157]
Brain Cancers	1. Mouse glioma model of GL261 2. Transgenic mouse with 4T1 mammary adenocarcinoma 3. Human brain metastasis tissues 4. Transgenic mouse models of lung and breast adenocarcinoma brain metastases	1. BAMs are evenly distributed in both naïve and glioma-bearing brains 2. LYVE1^+^MHCII^lo^CD206^hi^ PVMs are involved in the formation of a pre-metastatic niche around blood vessels during cancer metastasis 3. LYVE1^+^ PVMs may contribute to the breakdown of BBB during tumor metastasis to the brain 4. BAMs located in the meninges may play a role in facilitating cancer metastasis to brain-border structures	[44, 61, 171, 174, 176, 177]

### Biological features

As is widely known, in the steady state, microglia are highly ramified cells with multiple branches and processes extending from a small soma, actively moving to scan the entire brain parenchyma for detecting any homeostatic or pathological changes [81]. Upon sensing foreign or damage-associated factors, microglia transform from a ramified to an amoeboid shape, embodied in enlarged cell bodies and shortened cell processes. Amoeboid morphology reflects an activated or reactive state associated with phagocytosis and inflammatory functions regardless of whether these are protective or detrimental [13, 82]. Additionally, bipolar rod-shaped microglia have been observed during brain injury, probably related to aberrant neuronal circuits [83]. However, BAM subsets exhibit considerable variation in morphology. During homeostasis, the sdΜΦ and PVM subsets appear in a more elongated shape than microglia, and reside around blood vessels [9, 50, 84]. In contrast, the dmΜΦ are pleomorphic, mostly displaying a bipolar shape with many dendrites [50, 85]. Embryonic choroid plexus macrophages exhibit amoeboid morphologies with small processes [86, 87]. In adults, choroid plexus macrophages are characterized by their stellate shape and long, thin processes [50, 51, 85]. In inflammatory conditions, PVMs extend dendritic processes along the perivascular space in response to environmental chemotactic cues, and meningeal macrophages elongate their existing protrusions [88, 89]. Technical challenges, primarily due to their location in the ventricular system, have limited the observation of choroid plexus macrophages in the inflammatory status. The BAM pool also shows differences in motility, indicating differential adaptive functions among its subsets. PVMs, resembling microglia, exhibit limited motility, constantly extending and retracting their protrusions at the periphery of blood vessels [50, 88]. Similarly, meningeal macrophages display partial motility, using their processes to monitor the meningeal space [50, 84]. Of interest, the cpΜΦ show relatively stationary cell bodies but high motility in their processes which supports their role in immune surveillance. The cp^epi^ΜΦ, on the other hand, demonstrate substantial mobility in their cell bodies, allowing them to patrol the surface of the choroid plexus and closely survey their surroundings [90].

Several studies have outlined the molecular and genetic signatures of BAMs to distinguish them from other tissue macrophages [45, 48–50, 91, 92]. BAMs have been identified to express several pan-macrophage markers similar to those found in microglia, including CD45, CD11b, CSF1R, CD64, F4/80, MERTK, MHCII, CX3CR1, and IBA1 [8, 46, 50, 91, 93], making it challenging to discriminate between these populations. Immunological analyses, such as flow cytometry, have indicated that varying expression levels of certain markers could be used to differentiate between cell populations in studies [50]. For example, BAMs typically express higher levels of CD45 and MHCII compared to microglia [8, 50]. However, the use of CD45 levels as a reference is not always reliable, as some BAM subsets have been shown to express low levels of CD45 [91]. Moreover, under disease conditions, activated microglia exhibit an upregulation of CD45 and infiltrated monocyte-derived cells express high levels of both CD45 and CD11b, which complicate the characterization of BAMs [8, 94, 95]. Conversely, distinguishing microglia from other brain macrophage subsets can be more straightforward, as certain proteins, such as TMEM119 and P2RY12, are exclusively expressed by microglia [96, 97]. SIGLEC-H and SALL1 have also been identified as microglia-specific markers [91, 98, 99]. However, it is notable that the cp^epi^ΜΦ, a subset of choroid plexus macrophages originating from embryonic precursors, also express these markers and display a microglial transcriptome signature [49]. Additionally, while markers such as CX3CR1, TREM2, and CD33 are universally expressed in tissue macrophages, they have been extensively used in studies exploring microglial functions in both the steady-state and perturbed CNS [100–102]. Apparently, these investigations assessed a mixture of CNS macrophages rather than pure microglial populations. Now, both the anatomical compartment and morphology of BAMs have been included in their discrimination from microglia through immunohistochemistry methods [103]. BAMs can also be distinguished from microglia based on CD206 expression, which is extremely low in microglia under the steady-state condition [48, 50, 91, 104]. To further differentiate between BAM subsets, several surface proteins have been suggested, including CD38, MHCII, CCR2, and LYVE1 [91]. Most BAMs, approximately 75% of total cells, are CD38^+^ (or LYVE1^+^) MHCII^−^ subset [91]. The CD38^+^ (or LYVE1^+^) MHCII^+^ BAMs mainly make up subpopulations of sdΜΦ and PVMs [91]. The dmΜΦ predominantly contain single-positive MHCII^+^ BAMs and fewer CD38^+^ (or LYVE1^+^) MHCII^+^ subset [91]. Choroid plexus macrophages consist of three BAM subsets, each with a similar frequency: MHCII^−^, LYVE1^+^MHCII^+^, single-positive MHCII^+^. Furthermore, MHCII^+^ BAMs, located in the choroid plexus and dura mater, uniquely express CCR2, suggesting a monocytic origin [91, 105].

In terms of transcriptional profiles, previous reports described microglial signature genes including *Tmem119*, *Siglech*, *Slc2a5*, *P2ry12*, *Sparc*, *Fcrls*, *Olfml3*, *Sall1*, *Hexb*, and *Trem2* [49, 50, 54, 91, 106]. However, genes such as *Fcrls*, *Hexb*, and *Trem2* show comparable expression in certain BAM subsets [46, 49]. As previously mentioned, the cp^epi^ΜΦ subset exhibits significant transcriptional similarities with microglia [49]. Therefore, genes like *Sall1*, *Sparc*, *Siglech*, *P2ry12*, and *Tmem119* are more reliably considered microglial core signatures [49, 54, 107]. Yet, in mouse brains, *Sall1* expression has been observed in certain neurons and other glial cells, including astrocytes and oligodendrocytes [108]. During pathological conditions such as MS and AD, none of the aforementioned signature genes, except *Hexb*, have been found to be consistently expressed in DAM, nevertheless [107, 109]. The *Hexb* locus has been suggested for use in genetic manipulation and fate mapping of microglia, but not for BAMs, in the CNS [109]. Several sets of genes have been identified as comparatively specific to BAMs over microglia, including those expressed universally across different subsets or uniquely in a specific subpopulation. Common core genes of BAMs, apart from cp^epi^ΜΦ, include *Cd206* (*Mrc1*), *Cd163*, *Cd169*, *Cd36*, *Apoe*, *Ms4a7*, *Ms4a6c*, *Stab1*, *Lyz2*, *Pf4*, *Cbr2*, and *Tgfbi* [46, 49, 54]. The transcriptome of PVMs includes genes such as *Mrc1*, *Cd163*, *Lyz2*, *Pf4*, and *Lyve1* [47, 50, 106, 110]. Signature genes enriched in the sdΜΦ include *Lyve1*, *P2rx7*, *Ccr1*, and *Egfl7*, while those in choroid plexus macrophages include *Ccnd2*, *Ttr*, and *Lilra5* [49]. Furthermore, in the aging and inflamed CNS, the phenotype and transcriptional profiles of BAMs are adaptively altered but remain distinguishable from other myeloid cell populations through the use of high-dimensional mapping techniques, such as mass cytometry [91, 92, 104]. The utilization of different combinations of myeloid cell makers, including CD45, Cx3CR1, MHCII, CD11c, CD38, CD44, CD206, CD169, CD43, Ly6C, SIGLEC-H, and CD14, allows for more specific definition of cellular subsets [91, 92, 104].

### BAMs in Alzheimer’s disease

The anatomical position of BAMs at CNS borders, along with their biological features, confers upon them fundamental functions such as waste clearance, nutrient uptake, antigen recognition and presentation, and regulation of BBB permeability [110, 111]. In AD research, the focus primarily centers on microglia-mediated phagocytic clearance of amyloid β (Aβ) aggregates, yet the impact of BAM subsets on AD progression is less understood. AD, the most common brain disorder causing senile dementia, is characterized by permanent neuronal damage due to excessive extracellular masses of Aβ peptides and intracellular bundles of fibrillar Tau protein [8, 13]. Abnormal deposition of Aβ peptides in cerebral blood vessels can lead to cerebral amyloid angiopathy (CAA), a typical pathological feature of AD [112, 113]. Interestingly, early research using the TgCRND8 mouse model of AD revealed that stimulating PVM turnover, rather than microglial or astrocytic responses, could reduce CAA load, implying a pivotal role for PVMs in CAA progression [114]. Subsequently, it was discovered that PVMs contribute to Aβ-induced cerebrovascular oxidative stress by highly expressing the reactive oxygen species (ROS)-producing enzyme NOX2 and CD36, an Aβ-binding scavenger receptor [115, 116]. In Tg2576 transgenic mice, a model of AD, the deletion of CD36 through PVM repopulation reduced ROS induction and ameliorated neurovascular damage induced by Aβ deposition [117]. Additionally, depleting PVMs with clodronate, a type of chemicals known as bisphosphonates, suppressed ROS production and alleviated Aβ-induced cerebrovascular dysfunction [118]. A recent study indicated that the interaction between PVMs and microglia regulates microglial ability to engulf neuronal synapses during the early onset of AD [78]. PVMs secrete large amounts of SPP1, which promotes microglial expression of phagocytic markers such as C1QA, GRN, and CTSB, leading to aberrant engulfment of synapses [78]. In AD mouse models, the deletion of *Spp1* contributed to a reduction in synaptic loss [78]. In addition, ApoE4, the strongest genetic risk factor for late-onset and sporadic forms of AD, has been found to be associated with neurovascular alterations and BBB breakdown [119–121]. ApoE4 carriers show more severe dysregulated cerebral blood flow and cognitive impairment compared to non-carriers [122]. Accordingly, while ApoE4 has been proven to be functionally related to microglia, astrocytes, and oligodendrocytes, its sources and targets may also be linked to PVMs in the brain [123–125]. Future studies investigating the conditional deletion of *Apoe4* in PVMs may provide insights into the neurovascular pathologies associated with AD. Importantly, the development of new Cre transgenic mice, capable of differentially targeting parenchymal microglia and Lyve1^+^ PVMs, will be instrumental in elucidating the role of PVMs in AD progression [47].

### BAMs in Parkinson’s disease

PD is a well-known neurodegenerative disease characterized by movement deficits and autonomic dysfunction [126]. The neuropathological hallmarks of PD include the loss of dopaminergic neurons in the substantia nigra pars compacta (SNpc) and the formation of intraneuronal protein aggregates, known as Lewy bodies and Lewy neurites, primarily composed of insoluble alpha-synuclein (α-synuclein) [126]. α-Synuclein is mainly expressed in neurons and, to a lesser extent, in astrocytes, microglia, and macrophages [127]. Normally, α-synuclein exists as a soluble monomer, but under cellular stress, it can aggregate into insoluble forms such as oligomers, protofibrils, or fibrils [128]. Misfolded α-synuclein not only directly causes neurotoxicity resulting in neuronal death, but also activates immune cells that beget neuroinflammatory lesions. Microglia have been observed to exacerbate α-synuclein-mediated cerebral pathology by facilitating cell-to-cell transmission of α-synuclein through their exosomes [129]. A recent study has indicated that BAMs, not microglia, play a crucial role in α-synuclein-related neuroinflammation, through acting as antigen-presenting cells to initiate a CD4^+^ T cell response [79]. MHCII has been proven pivotal in mediating the communication between antigen-presenting cells and α-synuclein-specific CD4^+^ T cells associated with PD [130, 131]. In an α-synuclein overexpression mouse model of PD, the conditional deletion of MHCII in microglia has shown no effects on α-synuclein-induced neuroinflammation, such as the infiltration of monocytes and T cells [79]. In contrast, the depletion of BAM subsets significantly reduced inflammatory processes, including microglial activation and the recruitment of Ly6C^hi^ monocytes and CD4^+^ T helper cells [79, 132]. Additionally, an in-depth analysis of the transcriptional profiles of PD-associated BAMs has revealed that only a few BAM subsets undergo proliferation, respond to IFN-γ, and exhibit early stages of activation [79]. Meanwhile, the majority of BAMs remain quiescent, reflecting the proportion of MHCII^+^ BAMs within the total BAM population [79]. Most BAMs express phagocytosis-related genes, such as *Cd68*. The “disease-activated BAMs”, however, express multiple genes involved in inflammation, antigen presentation, and immune cell recruitment and infiltration, including *Il1b*, *Itgax*, *H2-Aa*, *Cd80*, *Cd74*, *Cd274*, *Ccl5*, *Cxcl10*, and *Mmp14* [79, 132]. In human PD postmortem brain tissues, CD3^+^ T cells have been observed to be closely adjacent to CD68^+^ BAMs in the perivascular spaces [79]. Overall, these results highlight that, in the pathogenesis of PD, BAMs are indispensable for antigen presentation, T-cell activation, and the infiltration of inflammatory cells. Furthermore, a study employing a transgenic mouse model of PD indicates that meningeal macrophages might participate in the clearance of brain α-synuclein via the glymphatic system and meningeal lymphatic vessels [43, 133]. Taken together, the mechanisms by which distinct subsets of BAM regulate PD progression are worth further exploration and may offer new directions for PD therapy in the future.

### BAMs in Stroke

Nowadays, ischemic stroke is a leading cause of mortality and long-term disability worldwide [134]. Both clinical features and brain imaging are used in the diagnosis of ischemic stroke versus intracerebral hemorrhage [134]. Generally, occlusion of a cerebral artery leads to an ischemic stroke, manifested by a severely insufficient blood and oxygen supply to the brain parenchyma, which induces widespread neuroinflammation and neuronal death [135]. After the onset of ischemia, the structure and function of the BBB are progressively disrupted, followed by the influx of hematogenous fluid into the extravascular space, leading to the development of vasogenic edema [136]. During the acute phase of a stroke, pathological changes in endothelial cells and the production of ROS further exacerbate the permeability of the BBB [137]. Microglia are quickly activated within the first few hours and release a considerable amount of pro-inflammatory cytokines, which subsequently recruit peripheral immune cells into brain parenchyma [138]. Infiltrated immune cells, such as monocytes, macrophages, and neutrophils, further aggravate the dysfunction of the BBB [138, 139]. BAMs, especially perivascular macrophages, may contribute to the pathological progression following ischemia due to their location. It has been revealed that BAM subsets are involved in early immune responses and persist into the late chronic phase of stroke [140]. However, the similarity in phenotypes and transcriptional signatures among microglia, BAMs, and blood-borne macrophages has increased the difficulty of accurately identifying pure BAM populations. Nevertheless, several previous studies investigating the role of BAMs in ischemic stroke might provide valuable references for future research.

Using CD163 as a marker for BAMs, a study analyzed the transcriptome of sorted CD163^+^ brain macrophages 16 h after ischemia–reperfusion in a rat model. It was found that, post-ischemia, these CD163^+^ BAMs underwent a functional shift towards enhancing leukocyte recruitment and increasing BBB permeability through the induction of vascular endothelial growth factor (VEGF) [141]. Depletion of BAMs using clodronate liposomes led to reduced granulocyte recruitment and decreased permeability of leptomeningeal and cortical vessels 24 h after ischemia [141]. Similarly, in post-mortem brain tissues of ischemic stroke patients, CD163^+^ PVMs were observed to express VEGF strongly [141]. Another study demonstrated that in both human and rat stroke samples, CD163^+^ BAMs exhibited both local proliferation and migration to the brain parenchyma three days after ischemic injury [104, 142]. Additionally, RNA-sequencing results indicated that CD163^+^ BAMs in the brain parenchyma displayed pro-inflammatory phenotypes, with many pro-inflammatory genes, such as *Nos2*, being highly induced [104]. Furthermore, in a mouse model of ischemic stroke, CD206^+^CD169^+^ BAMs demonstrated pronounced proliferation in perivascular spaces after ischemia, followed by their accumulation in the brain parenchyma [104]. Notably, in mice four days post-stroke, as a subset of CD169^+^ BAMs translocated into the brain parenchyma, blood-borne MDMs began to occupy the perivascular area [104]. Recently, using a mouse model of middle cerebral artery occlusion (MCAO), a study examined the transcriptional profiles of brain cell subsets 24 h after stroke. Six distinct subsets of BAMs, based on core signature genes such as *Lyve1*, *Cd163*, *Mrc1*, and *Cbr2*, were identified [143]. Moreover, the proportion of BAMs among all cells was found to significantly increase post-ischemia in the mouse brain [143]. Of note, one particular BAM subset, mainly found in the MCAO group, expressed high levels of MHCII-related antigen presentation molecules (such as *H2-Aa*, *H2-Ab1* and *Cd74*), suggesting that BAMs may play important roles in modulating the adaptive immune response after ischemic stroke [143]. In addition, certain BAM subpopulations expressed genes associated with cellular oxidative phosphorylation, endocytosis, and immune cell recruitment, potentially contributing to aggravated neuroinflammation [143]. It has been proposed that peripheral administration of IL-13 in ischemic stroke could induce anti-inflammatory responses in both microglia and macrophages, leading to neuroprotection [144]. However, in the later chronic phases of stroke, the massive infiltration of myeloid cells from the bone marrow and the phenotypic adaptation of BAMs [46], present challenges in identifying BAM-specific regulations in disease progression. Therefore, more refined genetic models and tools will help resolve many unanswered questions about the role of BAMs in the resolution stage of stroke.

### BAMs in multiple sclerosis

MS is a chronic autoimmune disease that causes inflammatory demyelination in the CNS, leading to progressive and irreversible neurodegeneration [68]. It stands as the most common immune-mediated disorder, featuring extensive infiltration and activation of peripheral immune cells in the CNS [68, 145]. Pathologically, MS is marked by demyelination, gliosis, and the loss of axons and neurons [145, 146]. MS typically manifests in young adults and often follows a relapsing–remitting pattern in patients, leading to progressive physical and cognitive disability with aging [147]. Neuroinflammation is recognized as a key mediator of lesion formation in the CNS during both the acute and chronic phases of MS [68, 145, 148]. A large body of evidence suggests that activated microglia and T cells, which accumulate at MS lesion sites, are major contributors to the disease [148, 149]. Sustained inflammation, driven by aberrant activities of microglia and T cells, results in a multitude of molecular stresses that cause damage in BBB, neurons, and oligodendrocytes [150]. Nevertheless, the potential role of BAMs in MS has not been as widely recognized.

So far, a few studies have demonstrated that BAMs may be involved in each stage of MS and contribute to defining lesion types in the CNS [77, 150–156]. An early study found that in normal human brains, CD163^+^ macrophages were confined to CNS-border areas. However, in MS brains, these macrophages were primarily observed in acute active lesions and at the rims of chronic active lesions, whereas they were rare in chronic inactive lesions and the centers of chronic active lesions [150]. Recent single-cell analyses of human MS identified the immune cell landscape, revealing that several BAM subsets correlate with MS pathology [151]. A subset of BAMs, expressing signature genes such as *CD163*, *F13A1*, and *LYVE1*, was found to be abundantly enriched in active MS lesions, where they are closely associated with inflammation and antigen presentation [80]. This subset was notably scarce in non-active lesions and predominantly located near blood vessels [80]. Conversely, a cluster of BAMs, characterized by genes regulating stress response and oxygen levels, such as heat shock proteins (HSPs), was specially observed in perilesional areas. These areas appeared normal in axonal myelin but showed macrophage infiltration [80].

Furthermore, the assessment of immunological mechanisms in human MS samples is challenging, leading to a reliance on studies conducted in the animal model of experimental autoimmune encephalomyelitis (EAE) [152]. In EAE mice, depending on the in vivo imaging technique, it has been revealed that the functional phenotypes of both microglia and macrophages evolve and adapt to the local microenvironment during the formation and resolution of neuroinflammatory lesions [153]. Importantly, the leptomeninges are now recognized as a crucial factor in MS, as considerable immune cells have been found to infiltrate the leptomeninges in both MS patients and EAE mice, and lesions commonly form in cortical areas adjacent to these locations [154, 155]. Of interest, compared to the leptomeninges, the dural layer has shown significantly less immune cell infiltration during chronic EAE and MS [155]. In addition, macrophages in the dura mater have been found to be less efficient than those in the leptomeninges at autoantigen presentation and T cell activation, leading to a defective inflammatory process in the dural meninges [155]. Biopsies from patients in the early phases of MS have shown that leptomeningeal inflammation often co-occurs with cortical demyelination [156]. Additionally, in most human MS cases, the severity of cortical demyelination has been found to positively correlate with the extent of leptomeningeal inflammation [156]. In the EAE model, the sdΜΦ and PVMs in the leptomeninges, proliferate during disease onset, and participate in antigen presentation and the activation of autoreactive effector T cells, which then migrate into the brain parenchyma and trigger lesion formation [157]. Moreover, during the chronic phase of MS, while the population of sdΜΦ decreases, PVMs continue to proliferate [154]. Thus, distinct BAM subsets exert differential functions in MS, influencing the progression of neuroinflammation.

Currently, there are limited drugs that target both microglia and BAMs for treating progressive MS, characterized by a predominance of proinflammatory myeloid cells [158]. Lately, an investigation showed that inhibiting the insulin-like growth factor-1 (IGF-1) signaling pathway in both microglia and BAMs significantly reduced the severity of CNS inflammation in EAE mice [159]. The absence of IGF-1 signaling resulted in minor changes in microglia but remarkably altered the transcriptional profile of BAMs, underscoring their role in progressive MS [159]. Another study used PLX5622, a CSF1R inhibitor, to deplete both microglia and BAMs. It was found that PLX5622 treatment significantly delayed the onset of EAE, although it had no effect on the chronic progression of the disease [160]. In summary, therapeutic agents targeting brain-resident macrophages offer promising avenues for the treatment of MS and for neuroprotection.

### BAMs in brain cancers

Malignant brain tumors can generally be divided into primary tumors that arise in the brain, such as gliomas, and brain metastases (BrMs) from cancers such as non-small-cell lung carcinoma (NSCLC), breast cancer, and melanoma [73]. Brain cancers are typically lethal, with most patients having a very poor prognosis, exemplified by a median survival of less than two years for patients with glioblastoma (GBM), the most malignant primary brain tumor in adults [71]. Studies on gliomas and CNS metastases have unveiled that macrophages represent the most abundant stromal cell-type and comprise up to 30–50% of the tumor mass [71, 161]. In the CNS, tumor-associated macrophages (TAMs) are a heterogeneous population that includes brain-resident microglia, BAMs and MDMs, which together create an immunosuppressive tumor microenvironment (TME) [73, 162]. TAMs contribute to most hallmarks of brain cancers, including tumor growth, invasion, metastasis, angiogenesis, and immune evasion [71, 73, 162]. Moreover, TAMs affect therapeutic responses in patients and limit the clinical efficacy of most immunotherapies such as immune checkpoint inhibitor (ICB), by fostering a symbiotic interaction between tumor cells and the TME [163]. Although TAMs have been extensively studied during past decades, research specifying the functional features of different TAMs subtypes within tumor lesions has been inadequate. Until lately, advances in single-cell technologies have enabled the characterization of TAMs at the single-cell level, identifying subpopulations with distinct tumor-modulatory functions.

In gliomas and BrMs, TAMs outnumber other immune cell populations such as dendritic cells, T cells, natural killer (NK) cells and neutrophils [164, 165]. Remarkably, TAMs are more abundant in primary brain tumors than in BrMs [164, 165]. Microglia and MDMs predominate among TAMs, leading most research to focus on understanding the functions of these two populations [71, 73, 161, 162]. It has been revealed that tumor-associated microglia and MDMs display distinct spatial distributions and have incompletely overlapping functions in brain tumors [166–168]. However, despite their potential importance in tumor angiogenesis, invasion, and metastasis through the modulation of vascular integrity and function [169], the role of BAMs in brain tumors has been less frequently reported.

A recent study revealed that BAMs are evenly distributed in both naïve and glioma-bearing mouse brains, with a significant cluster of cells highly and specifically expressing BAM markers such as *Pf4*, *Dab2*, and *F13a1* [44]*.* However, no distinct cluster was identified exclusively in glioma-bearing brains. It appears that metastatic cancer cells, which must breach the CNS interface to colonize the brain, are likely to interact with BAMs. PVMs, situated around arteries and veins in the brain parenchyma, may facilitate the extravasation of cancer cells across the BBB and aid in the formation of pre-metastatic niches. In specimens of human brain metastases from breast cancer, NSCLC, and melanoma, significant intra- and peri-tumoral infiltration of brain microglia and macrophages has been observed [170]. These cells display phenotypes associated with enhanced phagocytic and pro-tumorigenic functions [170]. A subset of PVMs, characterized as LYVE1^+^MHCII^lo^CD206^hi^ macrophages, is found in various healthy tissues, including the brain [61]. Of late, this macrophage subpopulation has been shown to coordinate the formation of multi-cellular “nest” structures near blood vessels, which leads to reduced effectiveness of the chemotherapy, in a murine model of breast cancer [171]. The inhibition of Tie2 activity in a PVM subpopulation that expresses Tie2 and VEGFA has been shown to prevent breast cancer metastasis and improve the overall survival in animal models [172]. In addition, there is evidence suggesting that PVM activation via tenascin C signaling is critical for co-opting endothelial cells to create a pro-metastatic vascular niche, facilitating breast cancer colonization in the lung [173]. LYVE1^+^ PVMs in the lung have been shown to maintain vascular tone by interacting with vascular smooth muscle cells, thereby contributing to the metastasis of cancer cells through the bloodstream [174]. Furthermore, penetrating the BBB poses a major challenge for melanoma cells in establishing melanoma brain metastases (MBM). Proteases such as cathepsin-S, have been reported to be essential for BBB breakdown [175], with brain-resident PVMs potentially being an important source of these proteases [176]. Consequently, functional remodeling of PVMs located in the brain may be one of the requisites for the formation of brain metastases. In addition, brain metastasis to the dura and leptomeninges has also been observed in patients with melanoma, lung, and breast cancers [177]. Although currently the data on the definitive role of BAMs in these tumors is lacking, it is probable that meningeal macrophages and PVMs, due to their anatomical locations, are involved in the formation of metastatic niches and the modulation of the local TME.

## Conclusion

Over the years, the potential role of BAMs in CNS-associated diseases has often been overlooked. Indeed, the number of BAMs is much lower than that of microglial cells, which play critical roles in brain development and neuronal excitability. In the past, techniques to fully assess these myeloid cell populations were underdeveloped. Recent advances in lineage tracing and the utilization of genetic models have largely unraveled the puzzle of BAM ontogenesis, setting the stage for further exploration of their functions. Particularly, the use of multi-omics technology in studying diverse CNS diseases has unveiled that BAM subpopulations are intricately linked with the pathogenesis and disease progression. However, most current data addressing the specific functions of BAMs and microglia under disease conditions, struggle with the technical challenge of differentiating and exclusively interrogating these cells, partly due to their phenotypic overlap and similarities. Nonetheless, understanding the precise role of different BAM subsets in both neurodegenerative and neuroinflammatory diseases is crucial, as the development of novel targeted therapies depends on an in-depth understanding of how distinct immune cell populations contribute to the CNS neuropathology. Future research on the genetic or epigenetic manipulation of specific BAM subpopulations will be pivotal in pinpointing their roles in various diseases.

## Data Availability

Not applicable.

## Abbreviations

AD: Alzheimer's disease
Aβ: Amyloid β
α-synuclein: Alpha-synuclein
BAMs: Border-associated macrophages
BBB: Blood–brain barrier
BCB: Blood-cerebrospinal fluid barrier
BrMs: Brain metastases
CAA: Cerebral amyloid angiopathy
CNS: Central nervous system
CSF: Cerebrospinal fluid
cpMФ: Stromal choroid plexus macrophages
cp^epi^MФ: Choroid epiplexus macrophages
DAM: Disease-associated microglia
DIMs: Disease inflammatory macrophages
dmMФ: Dural macrophages
EAE: Experimental autoimmune encephalomyelitis
GBM: Glioblastoma
HSCs: Hematopoietic stem cells
HSPs: Heat shock proteins
ICB: Immune checkpoint inhibitor
IGF-1: Insulin-like growth factor-1
MBM: Melanoma brain metastases
MCAO: Middle cerebral artery occlusion
MDMs: Monocyte-derived macrophages
MHCII: Major histocompatibility complex class II
MPS: Mononuclear phagocytic system
MS: Multiple sclerosis
NK: Natural killer
NSCLC: Non-small-cell lung carcinoma
PD: Parkinson’s disease
PVMs: Perivascular macrophages
ROS: Reactive oxygen species
scRNA-seq: Single-cell RNA sequencing
sdMФ: Subdural/leptomeningeal macrophages
SNpc: Substantia nigra pars compacta
TAMs: Tumor-associated macrophages
TME: Tumor microenvironment
TREM2: Triggering receptor expressed on myeloid cells 2
VEGF: Vascular endothelial growth factor
